# Ecological Momentary Assessment of Person-Reported Outcomes in Diabetes: Unlocking Insights with Continuous Glucose Monitoring and the Potential for Precision Medicine

**DOI:** 10.1007/s11892-026-01618-5

**Published:** 2026-03-10

**Authors:** Dominic Ehrmann, Norbert Hermanns, Andreas Schmitt, Laura Y. Klinker, Bernhard Kulzer

**Affiliations:** 1 Research Institute Diabetes Academy Mergentheim (FIDAM), Johann-Hammer-Str. 24, Bad Mergentheim, 97980 Germany; 2 Department of Clinical Psychology and Psychotherapy, University of Bamberg, Markusplatz 3, Bamberg, 96047 Germany; 3 German Centre for Diabetes Research (DZD), München-Neuherberg, 85764 Germany; 4 Diabetes Centre Mergentheim (DZM), Diabetes Clinic, Theodor-Klotzbücher-Str. 12, Bad Mergentheim, 97980 Germany

**Keywords:** Ecological momentary assessment, Continuous glucose monitoring, Precision medicine, Mental health, Person-reported outcomes

## Abstract

**Purpose of Review:**

Diabetes self-management is accompanied by time-varying emotional and motivational challenges that impact mental health. These day-to-day fluctuations can be assessed via ecological momentary assessment (EMA), that allows the repeated sampling of psychosocial variables in everyday life. The benefits of EMA over questionnaires mirror the benefits of continuous glucose monitoring (CGM) over HbA1c. We describe the insights generated by combining EMA and CGM and highlight its potential.

**Recent Findings:**

Research shows that glucose levels can influence subsequent mood, stress, cognitive functioning, and symptom reporting, with nocturnal hypoglycemia and overnight glucose being particularly relevant. Studies demonstrated the importance of differentiating between subjective person-interpreted and objectively sensor-assessed glucose levels. N-of-1 analyses revealed relevant intraindividual differences in the association between glycemic and psychosocial parameters.

**Summary:**

Combining EMA and CGM can enhance our understanding of the dynamic relationship between glycemic and psychosocial variables, supporting precision medicine approaches for mental health in diabetes.

## Introduction

Research over the past 30 years has highlighted the importance of psychosocial factors for diabetes management [1–5] and has established the maintenance or improvement of mental health as an important therapy goal [6–8]. Person-reported outcomes (PROs), defined as any report coming directly from people with diabetes without third-party interpretation [9, 10], are key in understanding the impact of diabetes management on psychosocial variables (e.g. experiences, behaviors, attitudes/cognitions) and vice versa [9, 10].

Traditionally, PROs are measured using questionnaires that assess variables retrospectively over a certain period of time in the past (e.g. the last 1–4 weeks). However, in recent years, the methodology of Ecological Momentary Assessment (EMA) has gained popularity within diabetes research [11, 12]. EMA comprises different methods of ambulatory assessment that allow the repeated daily sampling of PROs and self-reported behaviors in participants’ everyday life [13]. Participants’ attitudes and behaviors can be assessed longitudinally over several consecutive days or weeks, often multiple times a day [13]. EMA offers a more “real-time” and in-the-moment assessment of variables of interest, thereby reducing recall bias (e.g. peak-end effects) and increasing ecological validity compared to questionnaires [13–16].

The rise of EMA in diabetes research [11, 12] may be attributed to three factors: First, improvements in digital technology and the widespread use of mobile technology have simplified the implementation of EMA protocols via smartphones [11]. Second, the advent of continuous glucose monitoring (CGM) has sparked an increasing interest in better understanding daily glucose management and excursions, which can be contextualized by EMA data. Third, recent calls for precision medicine [17, 18] and precision mental health in diabetes [19] have made it necessary to look beyond traditional approaches in gaining a better understanding of intraindividual and interindividual differences.

In this narrative review, we provide an overview of EMA studies in diabetes over the past 5 years, focus on the promising possibilities of the combination of EMA and CGM, and highlight the potential of this combination for precision medicine and mental health approaches.

## The Basics of EMA

For current EMA studies, personal or a study-specific Smartphone are often used to allow for daily prompting, usually via push notifications [11–13]. EMA prompts are typically developed through literature review, expert consultation, and pilot testing to ensure relevance, clarity, feasibility, and acceptability. Also, the EMA prompts should address PROs for which day-to-day or within-day variability can be expected and thus are acceptable for daily assessments (e.g. fear of hypos might change from day-to-day whereas fear of complications is likely to be stable construct). Examples include: “How stressed do you feel right now?” or “Have you eaten in the past hour?”. EMA items are validated by assessing their reliability both within a person and between persons, longitudinal factor analysis, completion rates, and associations with related questionnaires or other data sources [13, 20–24].

EMA is particularly useful when capturing dynamic, context-dependent processes – such as mood fluctuations, eating behaviors, or physical activity – whereas traditional questionnaires are useful when a retrospective summary assessment of more stable constructs – such as depression, therapy satisfaction – are needed. Combining both can yield complementary insights: EMA offers ecological validity and fine-grained temporal data; questionnaires give overarching summaries.

An EMA study creates data on at least two levels, (1) the person and (2) days. Analyzing EMA data therefore requires multilevel models to account for the repeated measurements within individuals across days (so-called “nested data”). This allows for both within-subject and between-subject analyses [25]. For example, a within-person analysis might examine whether an individual reports higher diabetes-related distress on days when they also report lower mood, capturing dynamic, day-to-day fluctuations. A between-person analysis, in contrast, could assess whether individuals who, on average, report higher levels of social support tend to show a weaker association between daily diabetes distress and daily mood across the study period. More generally, between-person analyses in EMA studies can be used to identify clusters of individuals who show similar patterns in their within-person associations, thereby highlighting subgroups that may benefit from tailored interventions [25].

## Why are EMA and Diabetes a Good match?

Diabetes self-management is a 24/7 task with varying demands and challenges [6, 26]. On a day-to-day basis, people with type 1 and type 2 diabetes have to make multiple decisions that are relevant for glucose control. Consequently, it has been shown that people with diabetes spend a considerable amount of time per day thinking about diabetes and self-management tasks [27, 28]. A study by Priesterroth et al. demonstrated that people with diabetes spent a mean of 77 min thinking about diabetes which translates to diabetes being present every 12–13 min (based on 16 h wake time) [29]. Furthermore, it is estimated that there are around 42 factors that influence glucose levels [30]. The importance and influence of each factor can vary within a day and between days, leading to glucose fluctuations. When using CGM, people with diabetes have real-time feedback on these fluctuations and the impact their decisions have on glucose levels (biofeedback), something that spot blood glucose measurements or HbA1c values cannot offer. Besides varying glycemic levels and self-management challenges, people with diabetes are also facing varying motivational and emotional challenges that can also differ from day to day [31].

With these time-varying characteristics and demands of diabetes and glucose management, the impact of diabetes on people’s mental health can also fluctuate over time. Using CGM combined with EMA, it is possible to capture the time-varying characteristics of daily glucose management, related situational and personal circumstances or aspects and these variables’ interdependencies – a level of detail and insight which would be impossible to achieve based on common HbA1c and questionnaires assessments. Thus, EMA is a great fit for diabetes as it can be better aligned with the temporal resolution of CGM data [12]. Repeated daily assessments using EMA and CGM enable the collection of intensive longitudinal data per person that allow the analysis of within-person changes and effects as well as between-person differences [20]. Associations between PROs and CGM-metrics across different time points within an individual can be calculated that allow an in-depth analysis of not only contemporaneous (e.g. mood and mean glucose on the same day) but also temporal (lagged) associations (i.e. mean glucose on Monday predicting mood on Tuesday). This provides a more detailed understanding of the dynamic interplay of PROs and glucose parameters within an individual on a daily level. For between-person analysis, it can be evaluated whether persons with a higher Time in Range (% 70–180 mg/dl, 3.9–10 mmol/l) across study days also report an overall better mood. By combining EMA and CGM, precision monitoring becomes possible [32].

As diabetes technology continuously evolves, as can be seen by automated delivery systems (AID), daily living with diabetes and the daily challenges of diabetes self-management will change as well. EMA has the potential to capture the day-to-day impact of modern diabetes technology and how the increasing automation of glucose management impacts (daily) burden and other psychosocial variables. On the other hand, the integration of diabetes technology has significantly shaped the application of EMA in recent years. While earlier studies often used masked CGM to avoid reactivity [33, 34], the widespread use of real-time CGM and AID systems now makes masking less feasible and introduces new considerations for interpreting subjective glucose perceptions [12]. As technology increasingly automates glucose management and predicts/prevents hypoglycemia, the subjective experience of burden may shift – from managing glucose levels manually to being confronted with real-time glucose values to managing trust in an AID system and dealing with, for example, alert fatigue. EMA methodologies will need to adapt to these evolving contexts, capturing both objective data and the changing psychological and behavioral responses to advanced diabetes technologies.

## Literature Search

To provide an overview of current EMA research, we searched PubMed and Google Scholar using the terms “diabetes mellitus” in combination with either “ecological momentary assessment”, “ambulatory assessment”, or “experience sampling” for studies published in the last 5 years. Our literature search identified 38 publications. Four of these were review articles [9, 11, 32, 35], six were study protocols [21, 36–40], and three addressed a general occupational context [41–43], leaving 25 original articles [22–24, 44–65]. The review articles, study protocols and original articles are summarized in Table 1.

**Table 1:** Characteristics of the reviewed literature

Authors	Topic	Population & sample size	Duration of ambulatory phase	EMA protocol	Glucose measurement used for analysis	Key findings
*Review articles*						
Muijs et al. (2021) [35]	Systematic review on the associations between glucose variability and mood	8 included studies with type 1 and type 2 diabetes	1 day		SMBG/CGM	• No clear evidence of an association between mood and glucose variability. • More research needed combining EMA and CGM.
Nam et al. (2021) [11]	Systematic review on EMA for health behaviors and contextual factors	10 included studies with type 1 and type 2 diabetes	4 days − 6 months	2–28 prompts/day	SMBG/CGM	• Associations between adherence (missed insulin boluses, glucose checks) and negative affect. • More calorie intake after increased psychological stress. • Issues with adherence in the morning for adolscents with type 1 diabetes.
Hermanns et al. (2022) [32]	Narrative review on precision monitoring in diabetes using EMA	na	na	na	na	• Combination of EMA and CGM can enable precision monitoring in diabetes, potentially leading to precision mental health approaches
Hermanns, Kulzer, Ehrmann (2024) [9]	Narrative review on the importance of PROs and the potential of EMA	na	na	na	na	• Large potential of EMA for the assessment of PROs in clinical trials. • Calculation of variability measures of PROs besides mean-scores. • Combination of traditional questionnaires and EMA
*Original articles*						
Polonsky & Fortmann (2020) [44]	Impact of Time in Range on Mood	219 adults with type 1 diabetes	14 days	1 prompt/day	Unmasked CGM	• More Time in Range during the day was associated with more positive mood ratings in the evening. • No associations of Time above Range and glucose variability with mood were found.
Yu et al. (2021) [45]	Predicting mental health risks through machine learning using passive sensing from interactions with a diabetes app	142,432 people with type 1 and type 2 diabetes	4 weeks	Passive data collection only	SMBG	• Moderate evidence for prediction of mental health risks (defined as either prescription of medication for mental health issues or mental-health related assessments and interventions). • Moderate evidence for the importance of emotional state during blood glucose checks and blood glucose values for the prediction.
Ehrmann et al. (2022) [24]	EMA-based Time with Distress as a new PRO measure	178 adults with type 1 diabetes	17 days	1 prompt/day	Unmasked CGM	• Time with Diabetes Distress and Glycemia-specific Distress expressed as the percentage of days with elevated distress due to diabetes, hyper- and hypoglycemia and glucose fluctuations. • Daily diabetes distress highly associated with exposure to hyperglycemia. • High reliability and validity of the new measures. • Evidence of higher associations of EMA with CGM parameters than for questionnaires with CGM.
Zhang et al. (2022) [46]	Identification of barriers to diabetes self-management via machine learning	31 young adults with type 1 diabetes	30 days	Event-contingent related to mealtimes	SMBG	• Nonadherence (missed mealtime SMBG or missed insulin boluses) was inferred by using EMA-data. • Larger role for demographics regarding non-adherence (social determinants of health). • Promising results of stress, mood and fatigue predicting nonadherence.
de Wit et al. (2023) [47]	Association between mood and glucose variability	18 adults with type 1 diabetes	14 days	6 prompts/day	Masked CGM	• Overall no association between mood and glucose variability. • Moderating role of nocturnal hypoglycemia in the association between mood and glucose variability.
Jin et al. (2023) [48]	Impact of sleep duration on next-day stress and affect	166 adults with type 1 diabetes	14 days	5–6 prompts/day	Masked CGM	• Average sleep duration of 7.2 ± 1.2 h. • Longer sleep duration was associated with lower general stress and lower negative affect the next day. • No significant associations with diabetes-specific stress and positive affect.
Pyatak et al. (2023) [49]	Impact of glucose levels overnight on next-day cognitive functioning and activity	166 adults with type 1 diabetes	14 days	5–6 prompts/day	Masked CGM	• Overnight glucose led to lower next-day functioning. • Overnight glucose variability and exposure to hypoglycemia was associated with poorer cognitive functioning the next day. • Overnight exposure to hyperglycemia was associated with more sedentary time the next day.
Shapira et al. (2023) [50]	Associations of positive and negative affect with glucose	32 teens with type 1 diabetes	14 days	4 prompts/day	SMBG	• Overall: negative affect was associated with higher odds of hyperglycemic values (> 250 mg/dl, 13.9 mmol/l) • HbA1c ≤ 8%: Positive affect was associated with more favorable glucose control. • HbA1c > 8%: Negative affect was associated with less blood glucose checks.
Singh et al. (2023) [22]	Reliability and Validity of EMA of Cognition	• 198 people from a community sample • 128 adults with type 1 diabetes	• 15 days • 10 days	3 prompts/day	No	• Testing of cognitivie functioning via EMA was reliable, both between-persons and within-persons, in people with type 1 diabetes and a community sample. • 20 to 25 completed EMA prompts are needed to achieve stable reliability between and within persons. • Construct validity of cognitive EMA was established in both samples.
Søholm et al. (2023) [23]	Psychometric properties of the Hypo-METRICS app to assess the impact of hypoglycemia	• 64 adults with type 1 diabetes • 36 adults with type 2 diabetes	10 weeks	3 prompts/day	No	• Internal consistency was adequate and test-retest reliability was high. • Multi-level confirmatory factor analysis demonstrated factorial validity. • Validity was established with expected correlations to other scales.
Søholm et al. (2023) [51]	Qualitiative data on the validity, feasibility, and acceptability of the Hypo-METRICS app	• 10 adults with type 1 diabetes • 8 adults with type 2 diabetes	10 weeks	3 prompts/day	No	• The Hypo-METRICS apps was perceived as relevant, understandable, and feasible. • Participants also experienced a small intervention effect regarding hypoglycemia awareness.
Bruckner et al. (2024) [52]	Development of a Healthy Living Index based on accelerometric and EMA data	99 people with and without elevated insulin resistance (no antidiabetic medication)	7 days	3 prompts/week	No	• Healthy Living Index (HLI) incorporated EMA-based adherence to a healthy eating guideline. • HLI scores were significantly different between insulin-resistant and insulin-sensitive participants.
Coombes et al. (2024) [53]	Pain perception after physical activity in people with diabetic neuropathy	10 adults with type 2 diabetes	7 days	5 prompts/day	No	• Greater symptom intensity after physical activity: aching, sensitivity to touch, burning, shooting, tingling
Divilly et al. (2024) [54]	Concordance between sensor-detected and person-reported hypoglycemia	• 276 adults with type 1 diabetes • 321 adults with type 2 diabetes	10 weeks	3 prompts/day	Unmasked CGM	• Only minor overlap between sensor-detected and person-reported hypoglycemia. • Nearly 2/3 of sensor-detected hypoglycemia were not noticed by participants. • Over 40% of person-reported hypoglycemia happed at glucose levels higher than the threshold of 70 mg/dl (3.9 mmol/l).
Ehrmann et al. (2024) [55]	N-of-1 analyses differentiating the influence of perceived vs. actual glucose levels on daily diabetes distress	• 193 adults with type 1 diabetes • 186 adults with type 2 diabetes	17 days	1 prompt/day	Unmasked CGM	• Differences between subjective interpretation (perception) and objectively CGM-measured glucose values. • Total sample: Subjective interpretation of glucose levels showed greater associations with diabetes distress than actual CGM parameters - in particular: interpretation of glucose variability. • N-of-1 analyses: When diabetes distress was more informed by the subjective interpretation of glucose, well-being at the 3-month follow-up was worse.
Helgeson et al. (2024) [56]	Associations of supportive and conflictual peer contacts with mood and self-management	167 adolescents with type 1 diabetes	8 days	8 prompts/day	No	• Associations between peer support and positive mood, as well as conflict and negative mood. • Conflictual contacts were associated with decline in self-management in lagged analyses.
Hernandez et al. (2024) [57]	Reliability and Validity of EMA-response time measures for emotional clarity	196 adults with type 1 diabetes	14 days	5–6 prompts/day	No	• Good reliability of response time measures achieved with 4 to 7 completed EMA prompts. • Mixed evidence for the validity of response time measures.
McAlister et al. (2024) [58]	Perceived stress as moderator of the association between physicial activity and glucose in people without diabetes	15 teens without diabetes	7–14 days	4 prompts/day on weekdays 7 prompts/day on weekend	Masked CGM	• Stress significantly moderated the effect of physical activty on glucose: Higher stress than usual + higher physicial activity than usual ◊ lower glucose. • Stress did not moderate the association of sedentary time with glucose.
McInerney et al. (2024) [59]	Qualitative and quantitative study on the feasibility and tolerability of a digital phenotyping approach	35 adults with type 2 diabetes 33 adults without diabetes	2 months	2 prompts/day	No	• High tolerability of the digital phenotyping • People with type 2 diabetes were more comfortable to share their digital phenotyping data with their healthcare provider
Merwin et al. (2024) [60]	Profiles of disordered eating in type 1 diabetes	83 adults with type 1 diabetes	3 days	1–2 times/hour	Masked CGM	• Based on DEP-R questionnaire: 4 classes of disorderd eating (Bulimia - Binge Eating - Overeating - Low Pathology). • Binge eating and insulin restriction differed between the 4 classes. • Highest mean 3-day glucose levels for “Bulimia” and “Binge Eating” classes.
Søholm et al. (2024) [61]	Impact of day and night hypoglycemia on daily functioning	• 274 adults with type 1 diabetes • 320 adults with type 2 diabetes	10 weeks	2 prompts/day	Masked CGM	• Differences between sensor-detected and person-detected hypoglycemia. • No significant associations of sensor-detected hypoglycemia with daily functioning. • Significant associations of daytime and night-time person-reported hypogylycemia with decreases in mood, energy and cognitive functioning. • Worse sleep quality associated with night-time person-reported hypoglycemia; worse memory associated with daytime person-reported hypoglycemia.
Zaremba et al. (2024) [62]	Factors associated with EMA completion rates in the Hypo-METRICS study	• 277 adults with type 1 diabetes • 325 adults with type 2 diabetes	10 weeks	3 prompts/day	Unmasked & Masked CGM	• Overall app completion rate: 91%. • Higher completion rates: older age, routine CGM use, greater time below range
Zuniga-Kennedy et al. (2024) [63]	Impact of nocturnal hypoglycemia, sleep quality and mood on next-day cognitive functioning	• 18 adults with type 1 diabetes	15 days	3–6 prompts/day	Masked CGM	• Slower next-day processing speed after nights with higher than usual nocturnal hypoglycemia. • No significant associations of negative affect and sleep quality with cognitive functioning.
Hermanns et al. (2025) [64]	Associations of somatic and mental symptoms with dysglycemia	• 192 adults with type 1 diabetes • 179 adults with type 2 diabetes	8 days	3 prompts/day	Unmasked CGM	• Negative daily associations with glucose: speech difficulties, coordination problems, confusion, food cravings. • Positive daily associations with glucose: thirst, urination, taste disturbance, itching. • Elevated symptom reporting in people with elevated depressive symptoms, elevated diabetes distress. • Highly idiosyncratic associations.
Saito et al. (2025) [65]	Psychosocial factors in dietary management of type 2 diabetes compared with healthy adults	• 20 adults with type 2 diabetes • 16 adults without diabetes	14 days	3 prompts/day + event-contingent before and after eating, after waking-up	Masked CGM	• Lapses in diet predicted 2-hour postprandial glucose • Type 2 diabetes: Dietary lapses were predicted by vigor, fatigue and cravings • Without diabetes: Dietary lapses were predicted only by fatigue • Both: Eating-out was associated with dietary lapse
*Study protocols*						
Pyatak et al. (2021) [36]	Overall design of the FEEL-T1D study to analyze the impact of glucose on cognitive functioning and emotion	• 200 adults with type 1 diabetes	14 days	5–6 prompts/day	Masked CGM	• Completed, for results see [48, 49]
Divilly et al. (2022) [37]	Overall design of the Hypo-METRICS study to investigate the impact of hypoglycemia on daily functioning and mood	• 350 adults with insulin-treated type 2 diabetes • 200 adults with type 1 diabetes with intact awareness of hypoglycemia • 50 adults with type 1 diabetes and impaired awarness of hypoglycemia	10 weeks	3 prompts/day	Masked CGM	• Completed, for results see [23, 51, 54, 61, 62]
Søholm et al. (2022) [21]	Validation protocol for the Hypo-METRICS application to analyse the day-to-day impact of hypoglycemia	• 350 adults with insulin-treated type 2 diabetes • 200 adults with type 1 diabetes with intact awareness of hypoglycemia • 50 adults with type 1 diabetes and impaired awarness of hypoglycemia	10 weeks	3 prompts/day	Masked CGM	• Completed, see [23, 51, 54, 61, 62] • Development of the Hypo-METRICS apps in three phases
Vetrovsky et al. (2023) [38]	mHealth intervention to increase physical activity in prediabetes and type 2 diabets	340 people with prediabetes	12 months	Just-in-time prompts based on accelerometric data	No	• Ongoing
Wooldridge et al. (2023) [39]	Daily functioning in type 2 diabetes	100 veterans with type 2 diabetes	14 days	6 prompts/day	CGM	• Ongoing
Nam et al. (2024) [40]	EMA-based individual risk factors for increased glucose variability in type 2 diabetes	200 adults with type 2 diabetes	14 days	4 prompts/day	Masked CGM	• Ongoing

## Previous Systematic Reviews of EMA Studies

In 2020, Muijs et al. specifically reviewed evidence on the associations between glucose variability and mood based on eight studies. They concluded that there was no clear evidence for an association. However, they also included non-EMA studies using retrospective questionnaires and the included studies had small samples and used older, masked CGM systems. Muijs et al. called for higher quality studies with longer ambulatory periods to assess the impact of glucose variability more accurately [35].

In 2021, Nam et al. published a systematic review of EMA-assessed health behaviors and diabetes outcomes, showing some associations between missed insulin boluses/glucose checks and negative affect, and the impact of psychological stress on increased calorie intake. The systematic review concluded that EMA protocols were acceptable to participants and highlighted the “potential clinical utility of EMA in providing more timely and individualized feedbacks” (p. 10) [11].

## Reliability and Validity of EMA

While aspects of reliability and validity of EMA were not mentioned in the review of Nam et al. [11], several subsequent studies report on these aspects. Søholm et al. even published a separate psychometric evaluation of their EMA app “Hypo-METRICS” that was designed to capture the impact of hypoglycemia on sleep quality, mood, affect, cognitive functioning and social functioning [23]. They demonstrated factorial as well as convergent and divergent validity and showed high test-retest reliability of their measures. Internal consistency was satisfactory at the between-person level. In qualitative analyses, they showed high feasibility and acceptability of their EMA app [51]. The mixed-methods study of McInerney et al. also demonstrated the feasibility and tolerability of a 2-month digital phenotyping study that also used a twice-daily EMA protocol. Interestingly, in their study, people with type 2 diabetes were more comfortable to share their digital phenotyping data with their healthcare providers than people without diabetes [59]. In our study regarding diabetes distress, we proposed the percentage of days spent with elevated distress (either general or glycemic-specific) as new EMA-based PROs. We found high reliability coefficients between 0.76 and 0.90 for these measures [24]. Singh et al. demonstrated high reliability and validity of cognitive tests conducted via EMA and showed that only 20 to 25 completed EMA prompts were needed to achieve stable reliability [22].

In summary, the reviewed studies suggest that EMA-based measures can assess time-varying PROs relevant for diabetes management with sufficient reliability and validity. Some studies have attempted to increase validity by adapting validated questionnaires to the specificities of daily assessment [21, 24, 36, 63].

## EMA Completion

EMA completion refers to the percentage of EMA prompts that have been completed by participants across the study period [66]. Several meta-analyses focused on EMA completion rates in different study populations, however without diabetes. In a meta-analysis across research fields and including 477 articles, Wrzus et al. found a mean completion rate of 79% and drop-out rate of 10.6% [66]. In populations with high symptom burden such as chronic pain, 85% completion rates were reported [67]. Even in populations with cognitive impairment, a meta-analysis found a mean completion rate of 74.4% [68]. In the reviewed studies of people with diabetes, Zaremba et al. reported a completion rate of 91% across a 10-week EMA period [62]; in our studies, we observed a 79% completion rate for a 17-day period [24] and a 71.6% completion rate for an 8-day period [64]; and in the FEEL-T1D study, completion rates of 88.4% [49] and 86.4% [48] were reported. Among teens with type 1 diabetes, Shapira et al. observed a completion rate of 72% [50].

In summary, completion rates of EMA studies in people with diabetes are comparable to those in other populations and can be considered sufficiently high for the collection of reliable data and the implementation of EMA in studies. Using advanced statistical methods such as multi-level modelling, missing values can be dealt with rather well [20] and completion rates > 70% seem sufficient to obtain meaningful results.

## Going Beyond One Mean Score: Intensity, Frequency and Duration

Similar to CGM, EMA data offers much more dense and comprehensive information about a participants’ mental state, personal experiences, behaviors or outcomes. With EMA protocols spanning several days or weeks, other parameters besides one overall score can be easily calculated. Instead of estimating one sum score reflecting the overall level of diabetes distress, for instance, we used EMA data to evaluate the intensity of distress on each day and calculated the number of days with elevated distress within a time period (frequency) as well as the number of consecutive days on which elevated diabetes distress was reported (duration) [24]. Thus, this EMA-based measure can reflect the daily hassles of diabetes management much more precisely and exhaustively than a questionnaire score. Consequently, we found higher associations of the EMA-based diabetes distress measures with CGM-metrics compared to a questionnaire-based diabetes distress measure [24]. Other parameters, such as the variability of mental states (e.g., mood) within or across days can also be calculated and offer relevant information on fluctuations or stability (e.g., mood swings, emotional stability) [15].

Certainly, with daily sampling, analysis strategies for EMA data often do not rely on the calculation of means at all but consider the raw values from each prompt separately. Thus, every prompt or each day is included in the analysis on the same or equal footing, with no special weighting given to any one day. This is in strong contrast to traditional questionnaires, in which specific days can stand out in an individual’s consideration of a summary “past weeks” rating (e.g., peak-end effect, recency effect) [14].

## Effect of Glucose on Psychosocial Variables

The majority of the reviewed EMA studies present analyses of the temporal associations or effects of glucose on next-day psychosocial variables. For instance, Polonsky & Fortmann have shown that a higher Time in Range over the course of one day was significantly associated with being calmer, more cheerful, energetic and relaxed as well as less irritable, less frustrated and in better overall mood in the evening of that same day [44]. Interestingly, they also found evidence that more Time above Range (% >300 mg/dl, > 16,7 mmol/l) rather than more Time below Range (% <70 mg/dl, < 3.9 mmol/l) was associated with lower overall mood. The relative importance of hyperglycemia over hypoglycemia has also been found in other studies [24, 55]. Here, increased Time above Range (% >180 mg/dl, > 10 mmol/l) was associated with higher levels of daily diabetes distress on the same day [24]. A similar pattern can be seen in the study by Shapira et al. with teens, who found that negative affect was associated with higher odds of hyperglycemic values (> 250 mg/dl, 13.9 mmol/l). By contrast, positive affect was only associated with more favorable glucose (more Time in Range, less variability) in teens with HbA1c values ≤ 8% [50].

Several studies have analyzed the impact of overnight glucose on mood, daily and cognitive functioning the next day. In the FEEL-T1D study, longer sleep duration was associated with lower stress and lower affect [48], while higher glucose variability and increased hypoglycemia during the night led to decreased cognitive functioning (e.g., shorter sustained attention) [49]. The negative impact of nocturnal hypoglycemia on cognitive functioning was later corroborated by Zuniga-Kennedy et al. [63] and Søholm et al. [61]. However, it has to be noted that Søholm et al. found that only person-reported and not sensor-detected hypoglycemia during the night was associated with decreases in next-day mood, energy and cognitive functioning in people with type 1 and type 2 diabetes [61]. Of course, waking up at night (due to perceived hypoglycemia) is more disruptive than an unnoticed nocturnal hypo event, but the same effects on mood and functioning were also observed with daytime person-reported hypoglycemia compared to sensor-detected [61].

The importance of nocturnal hypoglycemia was also highlighted in the study by de Wit et al. who found that after a nocturnal hypoglycemic episode, increased glucose variability over the day was associated with increased levels of anxiety, fatigue and depressive mood [47].

In the DIA-LINK studies of type 1 and type 2 diabetes, we analyzed the effect of glucose levels in the 120 min prior to symptom assessment on symptom reporting [64]. This analysis revealed eight diabetes symptoms that were sensitive to changes in glucose (speech difficulties, coordination problems, confusion, food cravings, thirst, urination, taste disturbance, itching). As a consequence, focusing on the occurrence of these symptoms may help to detect changes in glucose levels such as hypo- and hyperglycemia (see Table 1 for specific associations). Interestingly, people with elevated baseline levels of depressive symptoms and diabetes distress reported higher intensities on nearly all symptoms indicating the importance of mental health for the experience of diabetes.

In addition to affect and symptoms, EMA can also assess self-management behavior in real-time. For example, Merwin et al. (2024) and Saito et al. (2025) assessed eating behavior via EMA, allowing a better understanding of predictors of maladaptive eating and its glycemic consequences [60, 65]. Merwin et al. found that a bulimic eating pattern was associated with frequent binge eating, insulin restriction and highest mean glucose over a 3-day EMA period in people with type 1 diabetes [60]. Saito et al. found that EMA-identified lapses in diet predicted 2-hour postprandial glucose and that these lapses in people with type 2 diabetes can be predicted by vigor, fatigue and cravings [65]. Identified predictors via EMA may allow for more precise interventions preventing maladaptive eating episodes.

In summary, recent EMA studies demonstrate that psychosocial variables such as mood, stress, diabetes symptoms (e.g. thirst, urination) and cognitive functioning can be significantly influenced by preceding glucose values. There is particular evidence for the importance of overnight glucose levels and nocturnal hypoglycemia regarding worse functioning and overall mood.

## Differentiation Between Subjective and Objective Glucose Experiences

The combination of EMA and CGM can offer two perspectives on glucose management within individuals. With EMA, the subjective perception or interpretation of glucose values can be assessed, for example with help of rating scales to assess the burden of hypoglycemia or fluctuations from 0 to 10 on a given day [55] or via logging a perceived hypoglycemic event in the EMA app [61]. This person-reported, subjective perception can then be matched with the CGM-based measurement of the corresponding objective interstitial glucose level. Based on results from the Hypo-METRICS and DIA-LINK studies, researchers have stressed that the subjective perception of glucose must be distinguished from the objective sensor-assessed glucose [32, 54, 55, 61, 64].

As described above, Søholm et al. clearly showed that not the actual objective sensor-detected hypoglycemic event (< 70 mg/dl, 3.9 mmol/l) but a subjectively perceived hypoglycemic event – independent of day- or nighttime – was associated with this person’s mood, energy, sleep quality, cognitive functioning and fear of hypoglycemia [61]. In a further analysis, Divilly et al. demonstrated that there was only minor overlap between sensor-detected and person-reported hypoglycemia (65% of sensor-detected hypoglycemia were not detected by participants; 43% of person-reported hypoglycemia had sensor readings > 70 mg/dl/3.9 mmol/l). Divilly et al. concluded that person-reported hypoglycemia and sensor-detected hypoglycemia should be understood as different hypoglycemia outcomes (with differential associations) and therefore separately reported in clinical and research settings [54]. This has important implications for EMA as the results indicate that the impact and burden of hypoglycemia cannot fully be captured by only using CGM, but that the subjective experience of hypoglycemia has to be assessed as well. For clinical practice, the findings have implications regarding the definition of hypo awareness and the review of CGM downloads.

In our analysis, we observed a disconnect between subjective and objective glucose experiences: people with type 1 and type 2 diabetes felt highly distressed due to varying glucose levels on 46% and 37% of days during the EMA period, respectively; in contrast, the CGM-calculated coefficient of variation was 32% and 22%, respectively, and thus well within the clinical target of ≤ 36% [69]. Furthermore, subjective perceptions of glucose showed a stronger influence on daily diabetes distress than objective CGM metrics. Specifically, the appraisal of glucose fluctuations was the strongest predictor of daily diabetes distress, while objective glucose variability was not a significant predictor [55].

In summary, the results highlight the importance of the subjective perception of glucose management, that can be to some extent disjunct from actual glucose levels. Both Søholm et al. [61] and Ehrmann et al. [55] found evidence suggesting that the appraisal of glucose may be more relevant for mental health than CGM metrics. Certainly, we found that also objective CGM metrics, especially Time above Range (> 180 mg/dl, 10 mmol/l) were significant predictors of daily diabetes distress indicating the relevance of suboptimal glycemic management for mood states [55]. Thus, subjective and objective glucose experiences should be considered simultaneously as both are important but can have differential associations. In conclusion, these studies show that it is not all about the numbers, but also about the subjective interpretation of the numbers.

## Potential of EMA for Precision Medicine

The differential effects of subjective and objective glucose can also be seen in the N-of-1 approach in a recent study [55]. An N-of-1 approach involves within-person analysis of a single individual’s data over time, allowing for the identification of patterns and associations that are specific to that person. This is particularly useful in EMA studies, as the high-frequency, real-time data provide insights into how symptoms, behaviors, and contextual factors fluctuate and interact within individuals, supporting personalized interventions. For each person, the individual contributions of subjective and objective glucose to daily diabetes distress were calculated. This was achieved by specifying a multilevel model that included random intercepts and random slopes for the regression of subjective and objective glucose on daily diabetes distress, thus computing separate regression coefficients for every person. When daily diabetes distress was more strongly driven by the subjective interpretation of glucose, mental health at the 3-month follow-up was suboptimal (e.g., more depressive symptoms, more fear of hypoglycemia). In contrast, when daily diabetes distress was more strongly driven by the objective CGM metrics, mental health at follow-up was better but glycemic outcomes were suboptimal. Thus, it was concluded that it is important to look at the contributors to diabetes distress [55]. A similar N-of-1 approach was taken in showing that for each person different symptoms are more or less sensitive to preceding glucose levels [64].

When looking at individual associations between glucose and psychosocial variables, different intervention strategies can be derived [32]. For example, psychoeducational and cognitive-behavioral approaches may be more effective when diabetes distress stems more from the interpretation, whereas diabetological, glucose-oriented interventions may be better for addressing diabetes distress that stems from actual glucose levels and glucose management problems. Case vignettes of three people from the DIA-LINK study that further illustrate this approach can be seen in Hermanns et al. [32].

We therefore argue that by combining EMA and CGM, a more individual, personalized understanding of the complex interplay between glucose and psychosocial variables is possible. This can serve as the foundation for implementing precision medicine or precision mental health approaches by selecting different interventional strategies for different clusters of people.

The potential of EMA for precision medicine is also described in the systematic review of Nam et al., as they highlight the possibility of analyzing between-person associations while controlling for within-person idiosyncrasies [11]. Two studies showed the potential of applying machine learning to EMA data. Yu et al. used passive sensing data regarding interactions with a diabetes app to predict mental health risks [45]. Zhang et al. identified stress, mood and fatigue states as potential barriers to self-management and predicted suboptimal self-management from these factors [46]. Both studies show a potential application of EMA in the real-time monitoring of potential risk factors. Consequently, when a certain threshold in an EMA-assessed risk factor is exceeded on a given day, an intervention can directly be provided at that moment “just in time”. Thus, EMA can be used for deploying ecological momentary interventions (EMI), defined as treatments provided when needed and in a naturalistic setting [70]. A special form of EMI are just-in-time adaptive interventions (JITAIs). JITAIs are interventions that aim at providing “the right type/amount of support, at the right time, by adapting to an individual’s changing internal and contextual state” [71]. The combination of EMA and CGM could facilitate providing such JITAIs, by (1) triggering an intervention when a certain threshold is exceeded in everyday life (indicating a current problem or need) and (2) by providing insights into individual associations between glucose and psychosocial variables that help select the most effective intervention.

For developing EMIs and JITAIs in diabetes, a better understanding of the interplay between glucose and PROs are needed in order to have a theoretical basis for developing and selecting treatments. Furthermore, EMA-specific cut-off values of specific PROs have to be established to decide when an intervention should be triggered in daily life. The types of interventions, suitable within a digital application, have to be specified and tailored to the daily issues they should address. Lastly, rigorous evaluations have to demonstrate the efficacy of EMI – also compared to more traditional approaches. A blueprint for developing JITAIs is provided by Nahum-Shani and colleagues [71, 72].

## Limitations and Challenges of EMA

When using EMA in diabetes, its benefits have to be weighted against potential challenges or barriers. Firstly, daily assessments can be burdensome for people, either because they take too long, are too frequent or because the timing is disruptive. Thus, the specific EMA protocol (i.e., duration, timing, prompts per day, items per prompts) has to be carefully developed, also making use of a participatory design including people with diabetes to ensure that the right questions are being asked at the right time. Secondly, responsiveness needs to be considered in that people may increasingly reflect on certain aspects when asked on a daily basis, thereby changing their behavior or appraisal. Thirdly, technical issues can lead to loss of data but also to frustration of participants. Finally, EMA studies will create lots of data that take time to manage and analyze, making it challenging for clinicians. To address and overcome these challenges, the first step is considering whether EMA is suitable to answer the research/clinical question or whether questionnaires are better suited. EMA protocols should be developed within a participatory design and tested in a pilot phase to ensure feasibility, acceptability and technical stability. Furthermore, automation of analyses, also using artificial intelligence, may help to handle the intensive longitudinal data.

## Conclusions

There has been a steep increase in diabetes-related EMA publications in the last years showing the potential of this methodology in better understanding daily living with diabetes and the time-varying demands of diabetes self-management. These publications have shown that EMA-based PROs are capable of being reliable and valid to assess diabetes-specific variables on a daily level with appropriate feasibility and acceptability.

Studies using EMA in combination with CGM support that glucose levels can be an important driver of mood, stress, sleep quality and cognitive functioning (Table 1). Especially, the importance of Time above Range and nocturnal hypoglycemia was highlighted in several studies [47–49, 61]. This evidence can be directly used for clinical practice to better understand, for example, a person’s mood changes due to changing glucose levels. Further research is needed to better understand the impact of glucose variability on mood [35, 47], but there is evidence suggesting that it might not be CGM-measured glucose variability that is impacting mood but the subjective interpretation of glucose fluctuations [55]. This also has important practice implications showing that even in the absence of clinically relevant glucose variability, people with type 1 and type 2 diabetes spent a considerable amount of time worrying about fluctuating glucose. Thus, education and training on how to interpret glucose fluctuations may be needed. Furthermore, findings suggesting that the interpretation of glucose may impact diabetes distress more than the objective CGM metrics [55, 61] can inform consultations, helping clinicians to place greater emphasis on the person’s subjective experience and tailor support accordingly. Applications of EMA for clinical practice can also be seen in the potential of N-of-1 analyses which can serve as a foundation for a precision medicine approach [55, 64].

**Fig. 1:**
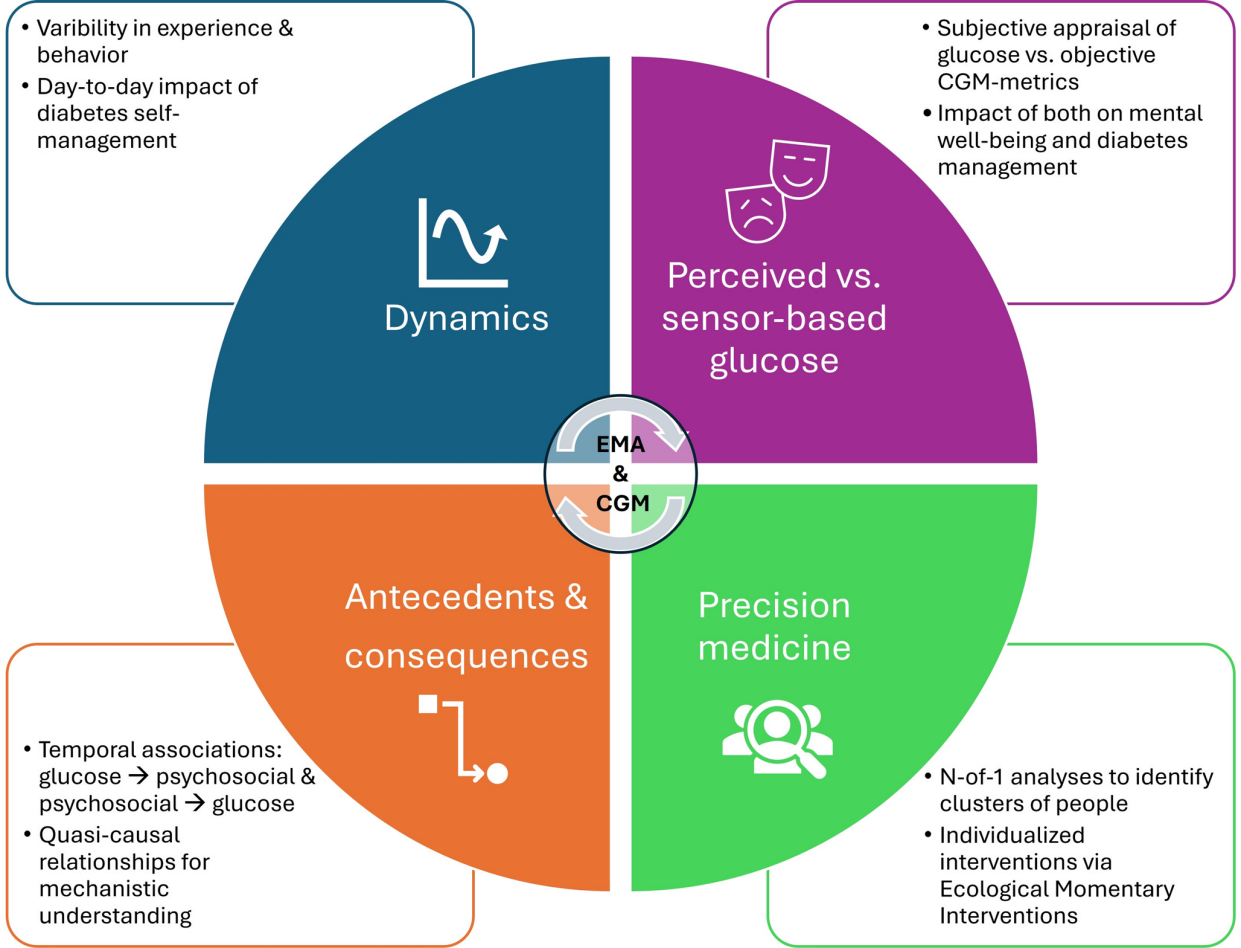
Potential of the combination of ecological momentary assessment and continuous glucose monitoring

In summary, the value and potential of EMA, particularly when combined with CGM, can be seen in four areas (Fig. 1):

1. Dynamics: Analyzing variability in experience and behavior, within a day and across several days. The fluctuation of self-management efforts can then be associated with psychosocial and glycemic outcomes. This allows a better understanding of the day-to-day impact of diabetes self-management efforts, the mental toll of diabetes self-management as well as identification of daily behaviors or treatment decisions that are associated with better outcomes.
2. Antecedents and consequences: Analyzing temporal relationships between psychosocial variables and glucose values that directly preceded them, or vice versa. This provides insight into quasi-causal relationships (i.e. one “causing” the other) and enables the identification of potential triggers, amplifiers or consequences. Such analyses could also distinguish between people for whom psychosocial well-being follows glucose levels, and people for whom glucose levels follow psychosocial well-being. These insights can then be used to tailor and select interventions.
3. Perceived vs. sensor-based glucose: Assessing the subjective perception of glucose levels and the objectively measured glucose. This allows a more comprehensive understanding of the challenges of glucose management. It also supports differentiating the cognitive-affective interpretation from the somatic state and helps to reveal mismatches and potential misinterpretations (e.g. high burden due to fluctuations vs. low coefficient of variation), which can be addressed through patient education or cognitive-behavioral strategies. This distinction may also become relevant with the increasing use of AID systems, where perception and trust may diverge from actual control.
4. Precision medicine: The intensive longitudinal EMA and CGM data per person allows the analyses of associations and patterns per person. The resulting insights can inform tailored interventions, support algorithms for JITAIs, and foster the development of digital health tools that integrate EMA and CGM data in real time. Conducting N-of-1 analyses can inform personalized care for the individual or clusters of people. Such approaches can enable the selection of interventions that are triggered based on individual risk patterns and are adapted to the individual’s association between glucose and psychosocial variables.

Next steps include establishing a platform that can integrate EMA and CGM data, generate automated analysis approaches for individualized pattern recognition, developing tailored interventions that can be deployed, and addressing challenges such as data protection and privacy, user adherence, and clinical feasibility. To fully realize the potential of EMA in diabetes and to achieve a precision medicine approach, interdisciplinary collaboration between behavioral scientists, clinicians, data scientists, technology developers and people with diabetes is essential.

## Data Availability

No datasets were generated or analyzed during the current review.

## Abbreviations & Acronyms

CGM: Continuous glucose monitoring
EMA: Ecological momentary assessment
EMI: Ecological momentary intervention
JITAI: Just-in-time adaptive intervention
PROs: Person-reported outcomes
